# Predictive model of spatial scale of forest fire driving factors: a case study of Yunnan Province, China

**DOI:** 10.1038/s41598-022-23697-6

**Published:** 2022-11-08

**Authors:** Wenhui Li, Quanli Xu, Junhua Yi, Jing Liu

**Affiliations:** 1 Faculty of Geography, Yunnan Normal University, Kunming, 650500 China; 2 Geomatics Engineering Faculty, Kunming Metallurgy College, Kunming, 650033 China; 3 GIS Technology Engineering Research Centre for West-China Resources and Environment of Education-Al Ministry, Kunming, 650500 China; 4 Center for Geospatial Information Engineering and Technology of Yunnan Province, Kunming, 650500 China; 5 Key Laboratory of Resources and Environmental Remote Sensing for Universities in Yunnan, Kunming, 650500 China

**Keywords:** Environmental sciences, Natural hazards

## Abstract

Forest fires are among the major natural disasters that destroy the balance of forest ecosystems. The construction of a forest fire prediction model to investigate the driving mechanism of fire drivers on forest fires can help reveal the mechanism of forest fire occurrence and its risk, and thus contribute to the prevention and control of forest fires. However, previous studies on the mechanisms of forest fire drivers have not considered the effect of differences in spatial scale of action of forest fire drivers on the predicted effect. Therefore, the present study proposes a spatial prediction model of forest fires that considers the spatial scale effect of forest fire drivers to predict forest fire risk. First, based on historical forest fire data and geographic environmental data in the Yunnan Province, geographically weighted logistic regression (GWLR) was used to determine the forest fire drivers and to estimate the probability of forest fire occurrence at locations where fire observations are absent. Then, multi-scale geographically weighted regression (MGWR) was used to explore the spatial scales of action of different drivers on forest fires. The results show that meteorological factors such as relative humidity, air temperature, air pressure, sunshine hours, daily precipitation, wind speed, topographic factors such as elevation, slope, and aspect, anthropogenic factors such as population density and road network, as well as vegetation type, were significantly correlated with forest fires; thus, they are identified as important factors influencing occurrence of forest fires in the Yunnan Province. The MGWR model regression results show that the role of different forest fire drivers on forest fire occurrence has spatial scale differences. The spatial scale of drivers such as altitude, aspect, wind speed, temperature, slope, and distance from the road to the fire point was larger and their spatial influence was relatively stable, with spatial heterogeneity having less influence on the model evaluation results. The spatial scale of drivers such as relative humidity, sunshine, air pressure, precipitation, population density, and vegetation type were smaller, and spatial heterogeneity had a more obvious influence on the model evaluation results. This study provides a reference for selecting drivers and evaluating their spatial scale effects to construct predictive regional forest fire models.

## Introduction

Forest fires are uncontrolled human-caused or natural (for example, from lightning strikes) events that burn forests, wastelands, and other vegetation in urban and rural areas. They create an important disturbance in the forest ecosystem and not only affect renewal of the forest but also threaten the safety of human life and property^1,2^. In recent years, forest fires have become increasingly serious as global climate has changed dramatically. According to statistics, the global forest cover loss due to forest fires is 1,190,000 hectares between 2001 and 2021, and in China, forest cover loss due to forest fires is 893,000 hectares during the same two decades. This shows that the high incidence of forest fires has resulted in a huge loss of forest resources. At the same time, the frequent occurrence of forest fires may further lead to soil erosion, increased land desertification and other adverse effects, ultimately destroying the ecological balance and affecting the human living environment. Therefore, how to effectively prevent the occurrence of forest fires and control their hazards is a challenge for forest management and governmental disaster prevention and mitigation departments around the world. Assessing forest fire risk by constructing predictive spatial models is important to prevent forest fires from occurring^3,4^. Among these, forest fire drivers are an important factor affecting the effectiveness of forest fire prediction in two main ways. First, the choice of forest fire driver has an impact on the model prediction results, meaning that different drivers may produce speculative results that are mechanistically difficult to interpret uniformly. The second aspect is that the expression of forest fire driver features affects the model prediction results. For example, expressing driver features at different spatial scales may result in predictions with different effects. Therefore, identifying forest fire drivers and their mechanisms is important for forest fire prediction.

Statistical and machine learning methods are the currently used methods to analyze the drivers of forest fires and their mechanisms^5,6^. These methods identify forest fire drivers and their influence through the learning of complex spatial relationships between forest fires and drivers; further, they provide spatial predictions of forest fires. The projection and mapping of the likelihood of regional forest fires and their dangers are then realized. Statistical methods include commonly used frequency ratios^7–9^, weight of evidence^10^ and multi-criteria decision analysis^11^. The basis of this type of method is to construct a relationship between historical fire data and the drivers, and use this relationship in combination with the domain knowledge of experts to analyze the contribution of each driver to forest fires. This type of model is more commonly used to determine forest fire drivers, analyze the mechanisms driving forest fires, and make spatial predictions of forest fires. However, a shortcoming of this approach is that the model itself has poor learning capability, weak error tolerance, and inability to handle errors. Therefore, the modeling results are not sufficiently accurate and the prediction results are poor. The second method commonly used to determine forest fire drivers and make spatial predictions of forest fires is the machine learning approach, using the common random forest model^5,12,13^, artificial neural networks^14^, logistic regression, among other methods^15,16^. The idea behind this approach is to combine artificial intelligence to learn the complex spatial relationships between forest fires and their drivers, identify the main drivers, and make spatial predictions of forest fires, mainly as adjustments to the model parameters to determine the effect of the drivers. The advantages of this method are that the model structure does not have to be pre-specified, unknown interactions can be handled, and in most cases, nonlinear functions can be handled with high explanatory power^17^. Therefore, machine learning methods have improved the fit of forest fire prediction models to a certain extent, resulting in better predictions^18^. However, this type of model requires a large amount of data for training samples during the modeling process.

The above analysis shows that statistical learning methods have poor learning capacity, are less tolerant, and less able to resolve errors. Using this type of method to determine forest fire drivers and make spatial predictions can result in poor prediction effectiveness and accuracy. By contrast, machine learning methods have a greater ability to learn and are more error-tolerant than are statistical learning methods. They can interpret data better and process data faster. Therefore, machine learning methods are widely used in forest fire prediction and driver analysis studies^19,20^. However, current research methods assume that the relationship between forest fires and their drivers is spatially identical everywhere, implying a stable spatial relationship. However, the geographical distribution of forest fires and their drivers is highly spatially heterogeneous due to differences and imbalances in the spatial distribution of the drivers, implying significant spatial non-stationarity in the relationship between the two. Spatial non-stationarity is generally defined as structural instability in the form of changing model parameters of the system^21^. Therefore, the current assumptions do not reflect detailed information on the spatial distribution of forest fires and their drivers, and are not conducive to detecting the driving mechanisms and spatial prediction of forest fires. Therefore, geographic weighted regression (GWR) models have been introduced into the study of forest fire prediction and factor driving mechanisms^22^. When this model was applied to the spatial analysis of forest fire drivers and spatial prediction of forest fires, it showed better prediction accuracy and had better fit than the global logistic regression model, which ignored the spatial non-stationarity of forest fires and drivers^22–25^. The GWR model proposed by Fotheringham et al. is an extension of the global regression model^26^. It adds spatial location information to the regression parameters to study the relationship between the independent and dependent variables within a certain range (bandwidth: Bandwidth is an important control parameter in the calculation of GWR model weights and can be divided into fixed and variable bandwidths. The bandwidth size directly determines the rate at which the weights decay with increasing distance: the larger the bandwidth, the faster the weight decay, and vice versa), which can effectively solve the problem of spatial non-stationarity. Therefore, more accurate identification of the spatial non-stationarity of forest fire drivers is a key issue in successfully analyzing the driving mechanisms and predicting forest fires.

However, a forest fire analysis model (geographically weighted logistical regression, GWLR) combined with GWR has certain limitations. Although they consider the spatial non-smoothness between forest fires and drivers in the modeling process and provide consistent modeling results with actual geographical phenomena, they assume that all processes operate at a uniform spatial scale (the GWR model behaves as an optimal bandwidth)^27,28^; that is, all drivers affect forest fires at the same spatial scale (action scale). This ignores the spatial variability in the scale of action of different drivers on the dependent variable, in that, there are some drivers whose effects on forest fires are likely to be global in space, whereas others have local effects and their scales of action are different^29^. Therefore, combining GWR models does not accurately identify the spatially non-stationarity relationship between drivers and forest fires. Scale is a key issue in all sciences^30^, especially in geographic information science^31,32^. In forest fire research, multiple classes of drivers (meteorology, topography, and anthropogenic activity) are often modeled to reveal complex underlying mechanisms. However, not all processes operate on the same spatial scale. Therefore, identifying spatial differences in the scales of action of forest fire drivers is key to more accurately characterizing spatial non-stationarity and resolving the driving mechanisms of drivers of forest fires. The current multi-scale geographically weighted regression (MGWR) proposed by Fotheringham in 2017 can overcome these limitations^28^. In contrast to the classical GWR model with its best bandwidth principle, MGWR allows different regression processes to operate on different spatial scales by providing independent bandwidths for the conditional relationships between the response variables and different prediction variables. At the same time, a specific bandwidth for each variable can characterize the spatial scale of their action on the predictive variables; thus, multi-scale geographically weighted regression methods can be used to identify the spatial scales of action of forest fire drivers and accurately detect spatial non-stationarity between drivers and forest fires to help resolve the mechanisms of driver influence on forest fires and the successful prediction of forest fires.

Based on the above background, this study proposes the introduction of a multi-scale geographically weighted model to identify the spatial differences in the scale of action of forest fire drivers to help characterize their spatial non-stationarity more accurately and to analyze the driving mechanism of each driver on forest fires. First, the historical forest fire data of the Yunnan Province from 2010 to 2020 were used as the basis, and topography, meteorology, vegetation, and population density were used as the main explanatory variables in the modeling and analysis. Second, the GWLR model was used to predict forest fires and identify forest fire drivers. Finally, MGWR was used to explore the spatial effect of forest fire drivers on the likelihood of forest fires in the Yunnan Province, providing a scientific basis for future forest fire modeling analysis and assessment.

## Results

### Multicollinearity and correlation test results

**Figure 1:**
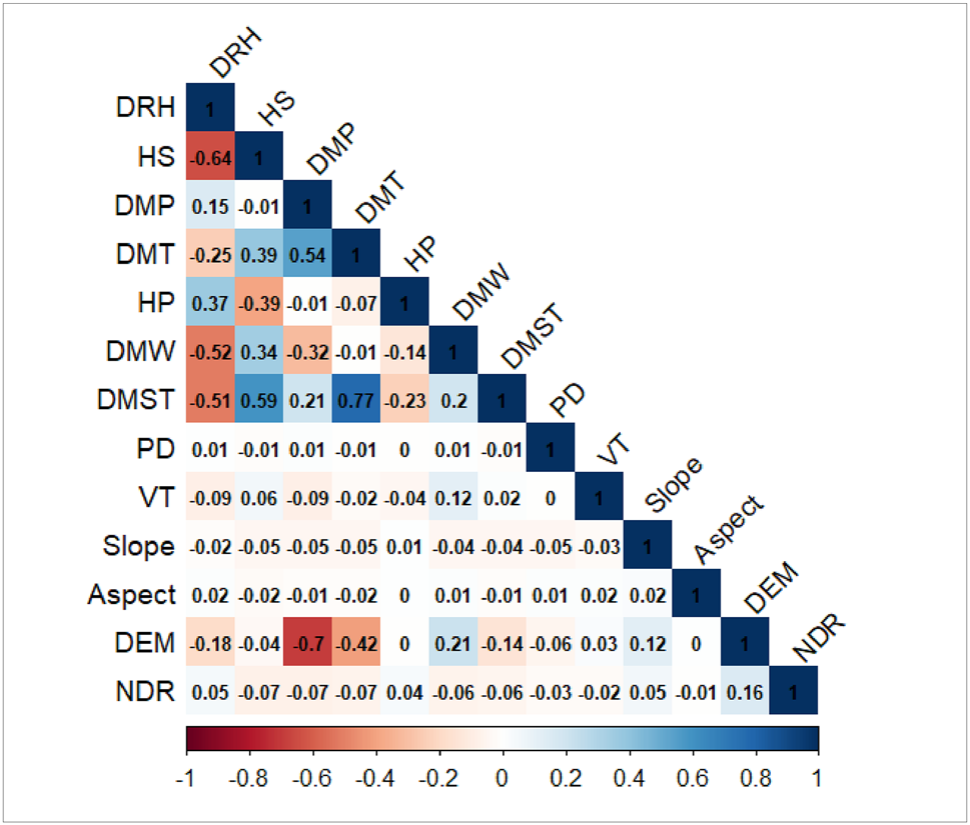
Results of correlation tests for candidate drivers. Daily relative humidity (DRH); 24 h of sunshine (HS); Daily maximum pressure (DMP); Daily maximum temperature (DMT); 24-h precipitation (HP); Daily maximum wind speed (DMW); Daily maximum surface temperature (DMST); Population density (PD); Vegetation types (VT); Slope; Aspect; Digital elevation model (DEM); Nearest distance from the road to fire point (NDR);

The results of the multiple covariance diagnostics show (Table 1) that when all candidate forest fire drivers were tested for covariance, the variance inflation factors (VIF) of the three meteorological factors, barometric pressure, air temperature, and ground temperature, were all above 10, and the tolerance (TOL) values were all below 0.1, indicating covariance between these factors and that each factor is a covariance between the highest, lowest, and mean values. By eliminating the six factors that had a high degree of collinearity, such as daily mean air pressure, daily minimum air pressure, daily mean air temperature, daily minimum air temperature, daily mean surface temperature, and daily minimum surface temperature, the remaining 13 candidate forest fire drivers were again validated. The final 13 variables passed the collinearity test (Table 1); in that, all VIF values were less than 10 and all TOL values were higher than 0.1. Finally, to verify the reliability of the collinearity test results, a correlation test was performed on the candidate drivers that passed the collinearity test based on the Pearson’s algorithm (Fig. 1). The results showed that the correlation coefficient between the surface temperature and air temperature was 0.77, suggestive of a strong correlation. Therefore, the surface temperature variable was eliminated, leaving a final 12 forest fire driver variables.

**Table 1:** Multicollinearity diagnosis results of model variables.

Serial number	Model variables	variable code	Before eliminating variables		After eliminating variables	
			TOL	VIF	TOL	VIF
1	Average daily relative humidity	DRH	0.102	9.806	0.417	2.398
2	24-h sunshine hours	HS	0.299	3.346	0.453	2.207
3	Daily mean atmospheric pressure		0.001	747.012	–	–
4	Daily maximum pressure	DMP	0.000	2805.608	0.368	2.716
5	Diurnal minimum		0.000	2683.704		–
6	Daily mean temperature		0.007	134.255		–
7	Daily maximum temperature	DMT	0.024	41.603	0.239	4.189
8	Daily minimum temperature		0.018	55.097		–
9	24-h precipitation	HP	0.771	1.298	0.793	1.261
10	Daily mean wind speed		0.635	1.576		–
11	Daily maximum wind speed	DMW	0.018	56.276	0.660	1.516
12	Daily mean surface temperature		0.074	13.575		–
13	Daily maximum surface temperature	DMST	0.021	48.606	0.250	3.994
14	Daily minimum surface temperature		0.989	1.011		–
15	Population density	PD	0.969	1.033	0.990	1.010
16	Vegetation types	VT	0.964	1.037	0.972	1.029
17	Slope	Slope	0.996	1.004	0.972	1.029
18	Aspect	Aspect	0.357	2.798	0.997	1.003
19	Altitude	DEM	0.783	1.278	0.401	2.494
20	Nearest distance from residential area to fire point	NDR	0.896	1.117	0.793	1.261

### Analysis of GWLR modeling results

#### Correlation analysis of driving factors and forest fire occurrence

**Figure 2:**
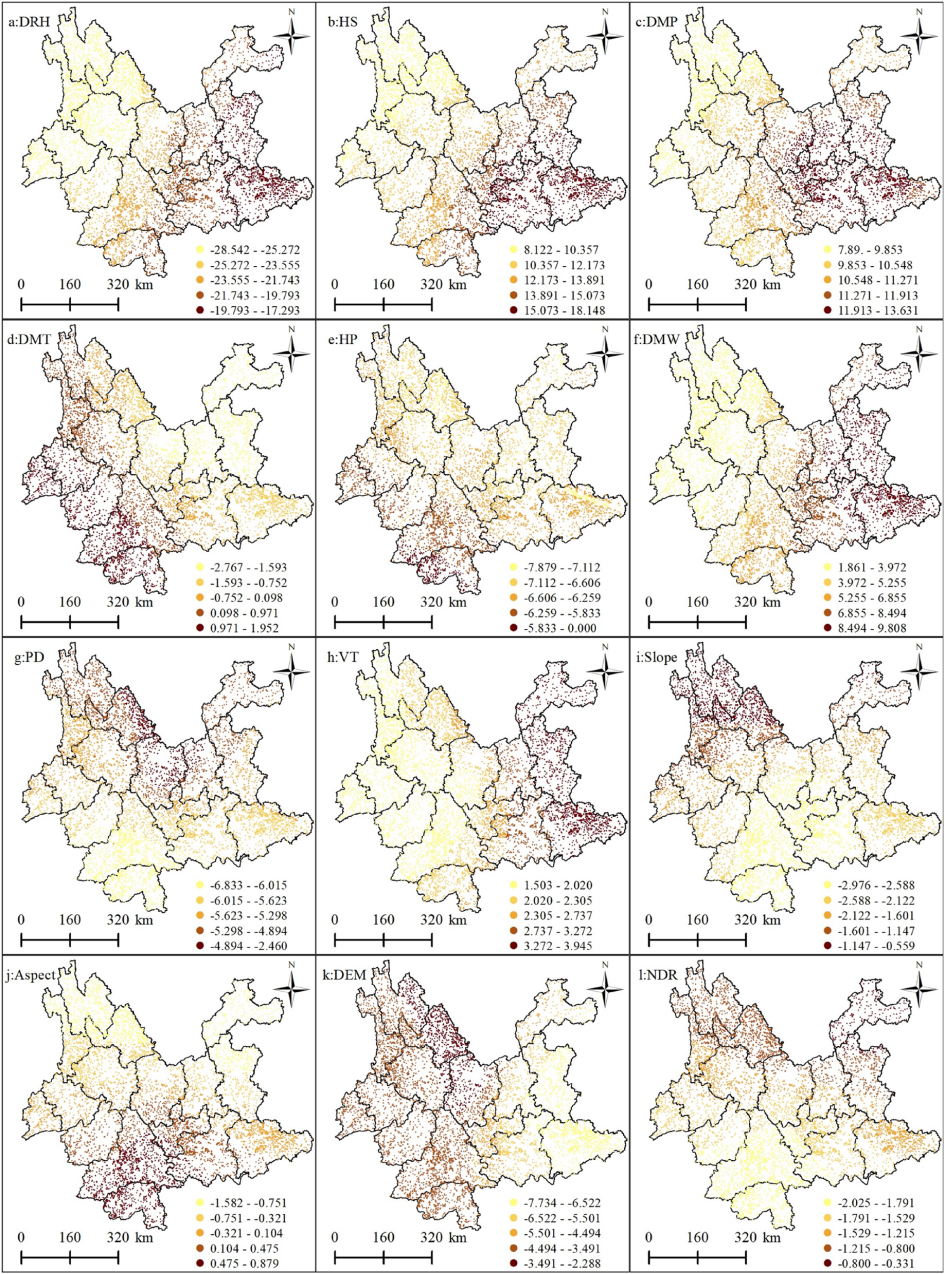
Significance plots for each explanatory variable based on the GWLR model t-test values. (**a**) Average relative humidity; (**b**) 24-h sunshine; (**c**) daily maximum pressure; (**d**) daily maximum temperature; (**e**) 24-h precipitation; (**f**) daily maximum wind speed; (**g**) Population density; (**h**) vegetation types; (**i**) slope; (**j**) aspect; (**k**) digital elevation model; (**l**) nearest distance from railway to fire point. Maps were generated by ArcGIS 10.8.12790 (https://www.esri.com/).

To determine the level of involvement of each variable in the model to help in the final determination of the main drivers of forest fires, the model significance threshold (t-test) was used as a reference. The t-test values for the GWLR model characterize the participation of a variable in the model results, with higher t-test values indicating higher implication in the model and more relevance, regardless of the positive or negative sign. A positive significance value indicates that the higher the value of the explanatory variable, the higher the probability of forest fires occurring, and vice versa. Conversely, when the value of t is negative, the value of the explanatory variable is inversely proportional to the probability of occurrence^24^. Figure 2 shows the participation of the candidate drivers in the GWLR-based forest fire prediction model and the correlation of each driver with forest fire after the collinearity test and correlation test. In this figure, 1.64 ≤|t|< 1.96 indicates significance at the 10% level showing that the driver is weakly correlated with forest fires and gives a weak explanation for them; 1.96 ≤|t|< 2.58 indicates significance at the 5% level, showing that the driver is moderately correlated with forest fires and gives a moderate explanation for them; |t|≥ 2.58 indicates significance at the 1% level, showing that the driver is significantly correlated with forest fires and gives a strong explanation for them. The results showed that the driving factors of relative humidity, sunshine, air pressure, wind speed, precipitation, population density, and elevation |t| are significantly correlated with forest fires, providing a strong explanation (Fig. 2a–c, e–g and k). Temperature, vegetation, and slope are moderately correlated, and slope direction and nearest distance from the road to the fire point have relatively low involvement and are weakly correlated with the explanation. Therefore, based on the GWLR modeling results, meteorological factors such as relative humidity, sunshine hours, air pressure, precipitation, and wind speed, topographic factors such as elevation, slope, and aspect; vegetation type, and anthropogenic factors such as population density and nearest distance from road to fire point were identified as the driving factors of forest fire occurrence in the Yunnan Province.

#### Spatial pattern analysis of the regression coefficients of the driving factors

**Figure 3:**
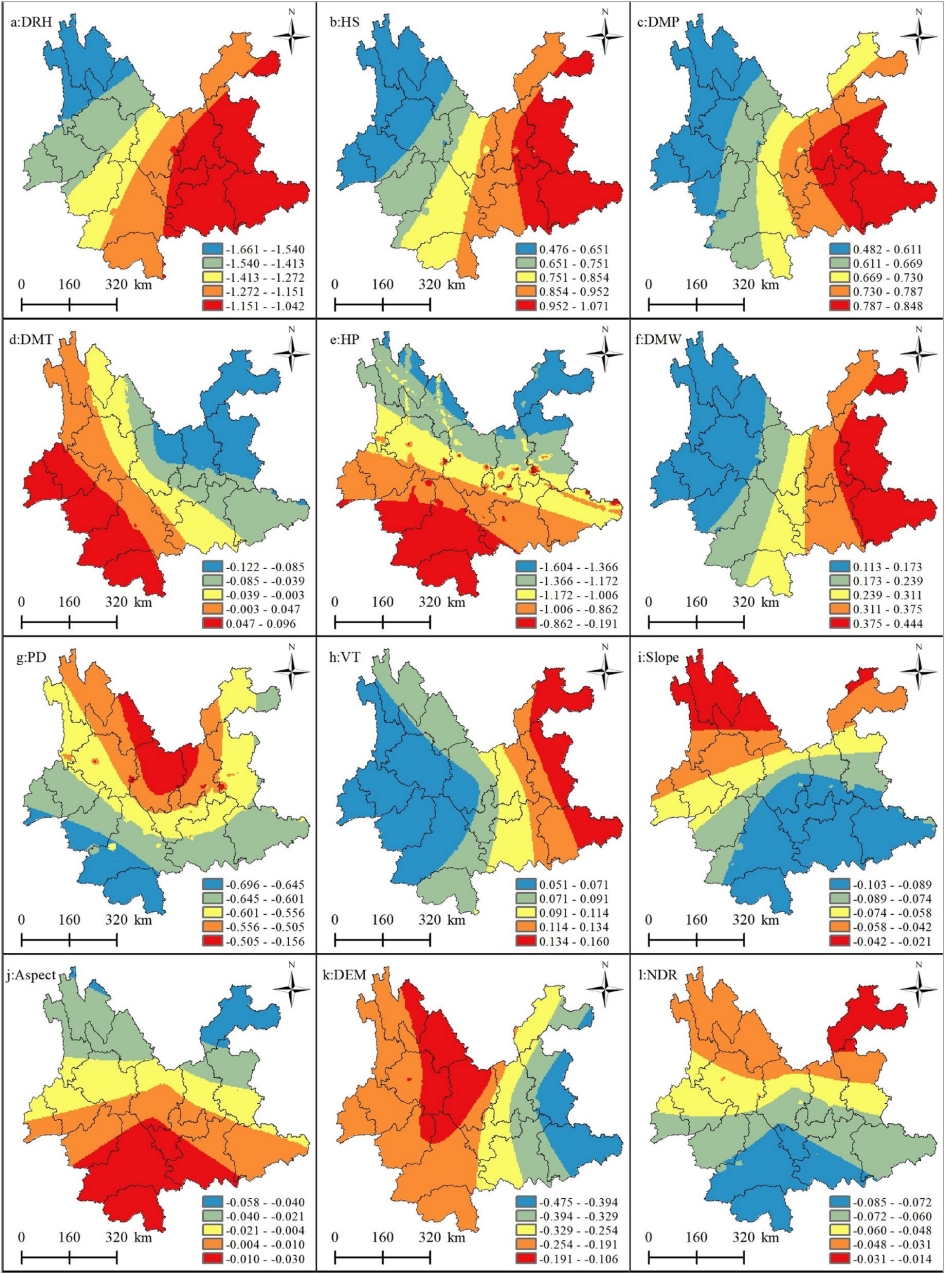
Spatial pattern of regression coefficients for the explanatory variables in the GWLR model. (**a**) Average relative humidity; (**b**) 24-h sunshine; (**c**) daily maximum pressure; (**d**) daily maximum temperature; (**e**) 24-h precipitation; (**f**) daily maximum wind speed; (**g**) Population density; (**h**) vegetation types; (**i**) slope; (**j**) aspect; (k) digital elevation model; (**l**) nearest distance from railway to fire point. Maps were generated by ArcGIS 10.8.12790 (https://www.esri.com/).

**Figure 4:**
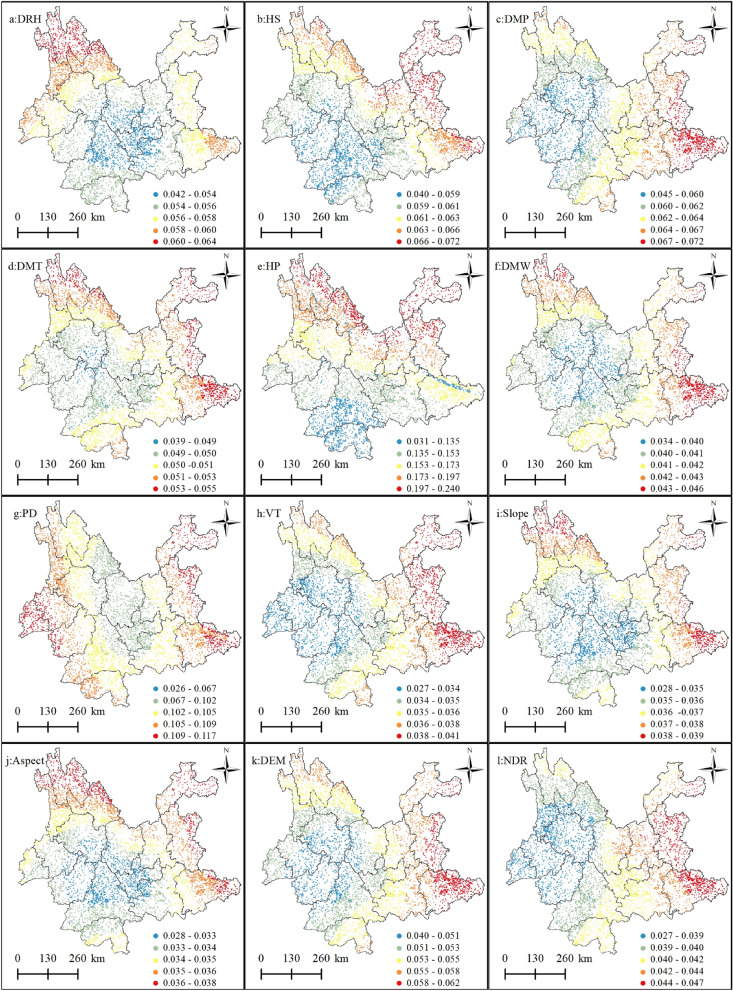
Spatial pattern of standard errors of regression coefficients for explanatory variables in the GWLR model: (**a**) average relative humidity; (b) 24-h sunshine; (**c**) daily maximum pressure; (**d**) daily maximum temperature; (**e**) 24-h precipitation; (**f**) daily maximum wind speed; (**g**) population density; (**h**) vegetation types; (**i**) slope; (**j**) Aspect; (**k**) digital elevation model; (**l**) nearest distance from railway to fire point. Maps were generated by ArcGIS 10.8.12790 (https://www.esri.com/).

Figure 3 shows the spatial mapping of the regression coefficients of the explanatory variables. To visually show the spatial non-stationarity of the individual variables, which describes the spatial variation in the relationship between the dependent and independent variables, the estimated coefficients of each driver were mapped by spatial interpolation using the Kriging interpolation tool of ArcGIS 10.8 software, and the regression coefficients of the drivers were displayed in a classification using the natural break grading method. This was done to help present the spatial heterogeneity of each driver's contribution to the probability of forest fire occurrence. Figure 4 shows the standard error of the coefficients for each driver, which was primarily used as a measure of their reliability. When the standard error value is small compared to the actual coefficient value, it indicates that the regression coefficient values estimated by the model are more reliable and vice versa. As shown in Fig. 4, the standard errors of the regression coefficients for all explanatory variables were smaller than the actual coefficient values (Fig. 3), indicating that the reliability of the regression coefficients estimated by GWLR is high.

Among the meteorological factors, the average daily relative humidity had a negative effect on forest fires, meaning that the probability of a forest fire decreases as the relative humidity increases. As shown in Table 2, the coefficient for mean relative humidity ranges from − 1.661 to − 1.024, with a mean value of − 1.293, indicating that the contribution of this driver to forest fires decreases by an average of 1.293 when the daily mean relative humidity value increases by 1; the data in the text are normalized, where "1" is the unit, as below. The coefficient also tended to decrease spatially from northwest to southeast (Fig. 3a). The modeling results showed that the probability of forest fire occurrence increases with increasing sunshine hours. The coefficient for sunshine hours ranged from 0.339 to 1.070 with a mean value of 0.797, indicating that an increase in sunshine hours increased the contribution of this driver to forest fire occurrence by an average of 0.797. The coefficient also showed a spatially decreasing trend from northwest to southeast (Fig. 3b). Air pressure and temperature also had positive effects on forest fire occurrence; the modeling results show that the coefficients for barometric pressure range from 0.362 to 0.668 and for air temperature from − 0.137 to 0.099. The coefficients tended to decrease spatially from east to west versus west to east (Fig. 3c, d). Precipitation had a negative effect on forest fires. The modeling results showed that the probability of forest fires decreased as precipitation increased. Its coefficient ranged from − 1.607 to 0, with a mean value of − 1.047. This indicates that an increase of 1 unit in sunshine hours reduced the contribution of this driver of forest fire occurrence by 1.047 (Table 2). Its coefficient tended to decrease spatially from north to south (Fig. 3e). Wind speed had a positive effect on forest fire occurrence, which indicates that, as the wind magnitude gradually increased, the probability of forest fire occurrence also increased. The wind speed coefficient ranged from 0.065 to 0.445 with a mean value of 0.251, indicating that when the wind speed increased by 1 unit, the contribution to forest fire occurrence increased by 0.251 (Table 2). The coefficient tended to decrease spatially from east to west (Fig. 3f).

**Table 2:** GWLR model regression coefficient statistics.

Variable	Mean	STD	Min	Median	Max
Average daily relative humidity	− 1.293	0.189	− 0.661	− 1.240	− 1.024
24-h sunshine hours	0.979	0.156	0.339	0.799	1.070
Daily maximum pressure	0.691	0.091	0.362	0.668	0.849
Daily maximum temperature	− 0.009	0.060	− 0.137	− 0.008	0.099
24-h precipitation	− 1.047	0.217	− 1.067	− 1.027	0.000
Daily maximum wind speed	0.251	0.103	0.065	0.228	0.445
Population density	− 0.586	0.058	− 0.697	− 0.591	− 0.066
Vegetation types	0.089	0.028	0.042	0.076	0.161
Slope	− 0.075	0.024	− 0.103	− 0.088	− 0.022
Aspect	− 0.005	0.020	− 0.059	− 0.003	0.030
Altitude	− 0.258	0.089	− 0.475	− 0.216	− 0.093
Nearest distance from road to fire point	− 0.060	0.015	− 0.086	− 0.064	− 0.014

The modeling results showed that vegetation type positively influenced the probability of forest fires. The coefficient range (0.042–0.161) indicates that vegetation types in the Yunnan Province may increase the probability of forest fires. For example, *Pinus yunnanensis* and planted eucalyptus forests are flammable because of their dominant species.

The terrain factor slope and elevation had a negative effect on forest fires, with the probability of forest fire occurrence decreasing as elevation increased. The coefficients ranged from − 0.475 to − 0.093 with a mean value of − 0.258, indicating that as elevation increased by 1 unit, its contribution to forest fire occurrence decreased by 0.258. The spatial distribution pattern of elevation coefficients in shown in Fig. 3k. The range of slope coefficients is − 0.103 to − 0.022, with a mean value of − 0.075, indicating that as the slope increases by 1 unit, its contribution to forest fires decreases by 0.075. The trend of slope orientation on forest fires is from north to south.

Regarding anthropogenic factors, the modeling results showed that the coefficient for population density ranged from − 0.697 to − 0.066, with a mean value of − 0.586, indicating that as population density increases by 1 unit, its contribution to forest fire occurrence increases by 0.586. The coefficient tends to decrease spatially from north to south (Fig. 3g), and the distance from the road to the fire site had a spatial distribution pattern of the coefficient decreasing from northeast to southwest (Fig. 3l).

#### Spatial distribution characteristics of forest fires based on GWLR

**Figure 5:**
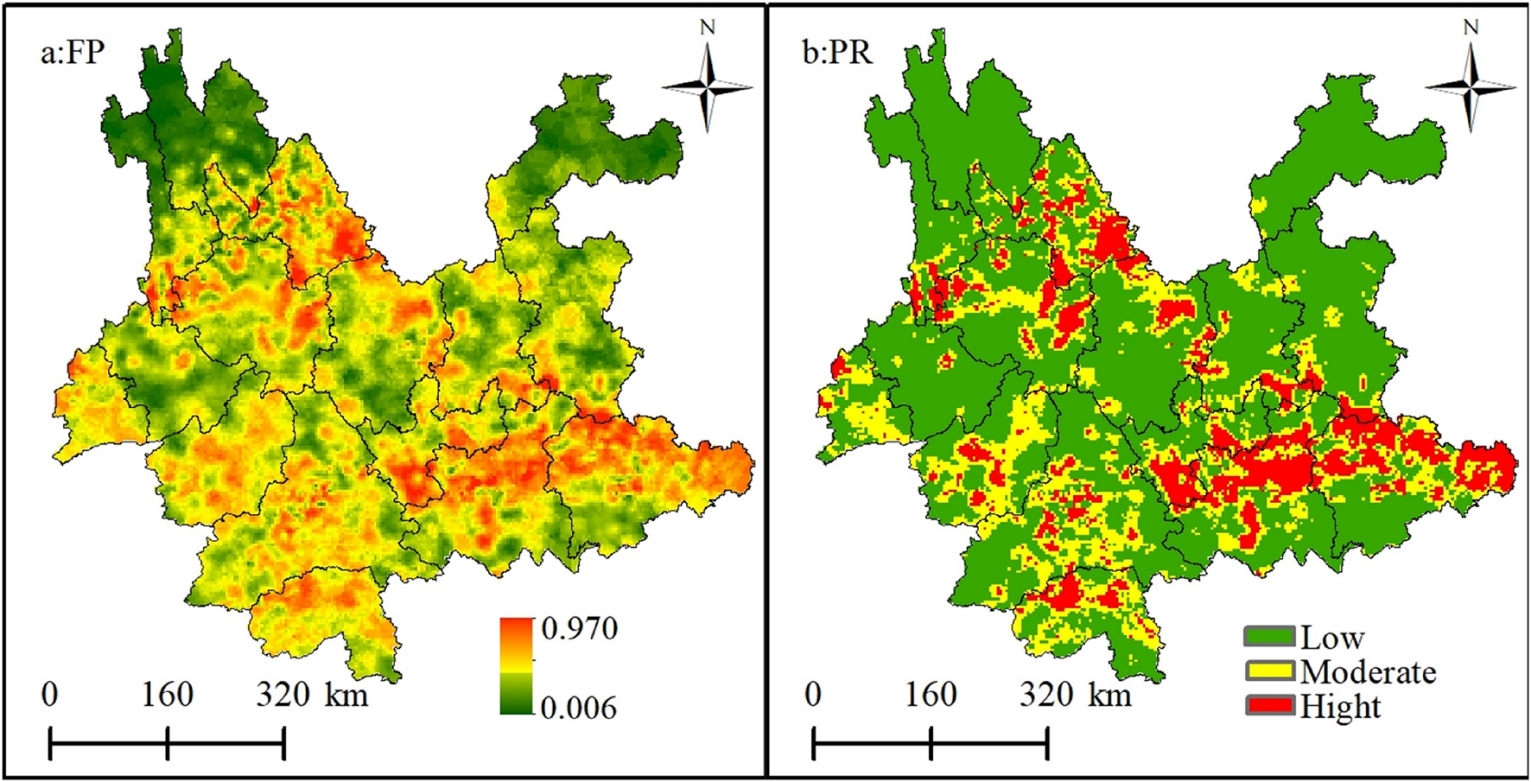
Probability distribution and classification of forest fire risk in the Yunnan Province based on GWLR models: model a is the predicted probability value; b is the fire risk level map. Maps were generated by ArcGIS 10.8.12790 (https://www.esri.com/).

Based on the GWLR modeling results, the spatial distribution of forest fire probability in Yunnan Province was interpolated and analyzed, using the kriging interpolation tool in ArcGIS 10.8, to produce the map shown in Fig. 5a. Based on the default threshold value of 0.5 and the optimal threshold value (cut-off) of 0.640 for the predicted probability of forest fire occurrence in the Yunnan Province, calculated using the Jorden index^33^, the GWLR model predicts that the probability value is greater than the critical value of fire occurrence and less than no fire occurrence. The value serves two purposes in the training sample set and the full sample set; first, to calculate the prediction accuracy based on the value to determine whether a forest fire occurs or not; and second, to zone the fire risk level for the Yunnan region. The GWLR model predicts that probability values (P) < 0.50 are classified as low fire risk class zones, 0.50 ≤ P < 0.640 as medium fire risk class zones, and P ≥ 0.640 as high fire risk class zones (Fig. 5b).

Figure 5 shows a clear geographical differentiation in the spatial distribution of forest fire risk probability and fire risk level in Yunnan Province. The high-risk areas are mainly distributed in the Honghe, Wenshan, and Lijiang states, with scattered distribution in Xishuangbanna, Nujiang, and Dali states. Second, the moderate risk areas are mainly distributed in the south and northwest of Yunnan province and its central region. Finally, the low-risk level areas are mainly distributed in Diqing, Zhaotong, Baoshan, and Chuxiong. The overall map of Yunnan Province shows that forest fires mainly occur in the southeastern, southern, and northwestern parts of the province, with the northwestern and central parts having a lower probability of forest fires and a lower fire risk level. Therefore, the focus of forest fire prevention and monitoring in Yunnan Province should be on Nujiang, Lijiang, and Dali in the northwest; Wenshan and Honghe in the southeast; and Pu'er and Xishuangbanna in the southwest. The reasons for this are as follows: first, the forest cover in these areas is larger, and the primary sector is the main economic sector, where agricultural production activities are carried out, leading to a higher probability of man-made fires; second, the types of forest vegetation in these areas are mainly coniferous forests, shrub forests, and other flammable tree species.

### Validation of GWLR model fit results

To more accurately identify the drivers of forest fires and analyze their complex spatial relationships with forest fires, this study evaluated the modeling accuracy and fit of the model, based on the area under the receiver operating characteristic (ROC) curve (AUC). The range of AUC values is generally between 0.5 and 1.0, with very weak accuracy when 0.5 < AUC ≤ 0.6, weak accuracy when 0.6 < AUC ≤ 0.7, moderate accuracy when 0.7 < AUC ≤ 0.8, high accuracy when 0.8 < AUC ≤ 0.9, and very high accuracy when AUC > 0.9. When the AUC is 0.5, the model has low accuracy and a poor fit. Table 3 shows the fit and prediction accuracy of the GWLR model for the three sample groups and the full data sample. The AUC value was 0.902 and the prediction accuracy was 82.9%, indicating that the model fit and prediction accuracy were both good. Therefore, the forest fire drivers identified and the correlation results between them are reliable.

**Table 3:** Evaluation indicators for GWLR results.

Sample	Model	AUC	Cut-off value	Accuracy /%	
				60%Training sample (%)	40% Training sample (%)
Sample 1	GWLR	0.906	0.671	81	79.1
Sample 2	GWLR	0.907	0.674	80.5	79.8
Sample 3	GWLR	0.902	0.663	80.7	80.1
Complete dataset	GWLR	0.902	0.640	82.9	

### MGWR-based variability analysis of the spatial scale effects of forest fire drivers

**Figure 6:**
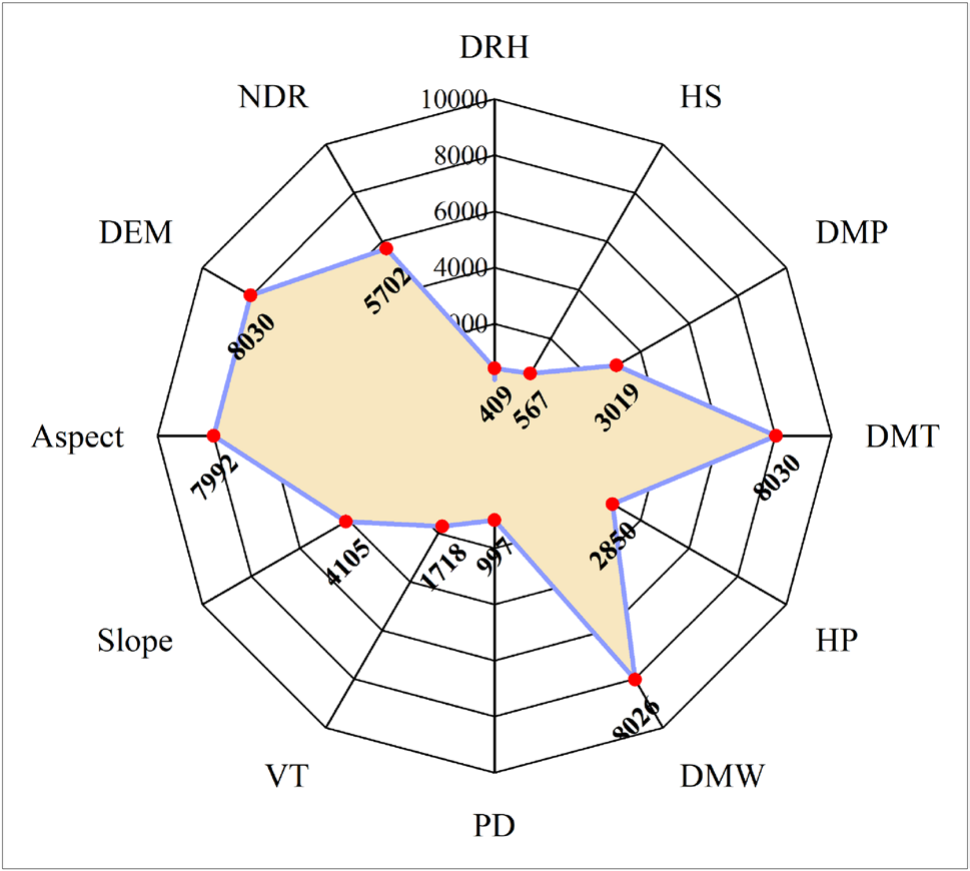
Spatial scales of action of individual forest fire drivers. Daily relative humidity (DRH); 24-h sunshine (HS); daily maximum pressure (DMP); daily maximum temperature (DMT); 24-h precipitation (HP); daily maximum wind speed (DMW); population density (PD); vegetation types (VT); slope; aspect; digital elevation model (DEM); nearest distance from the road to fire point (NDR);

**Figure 7:**
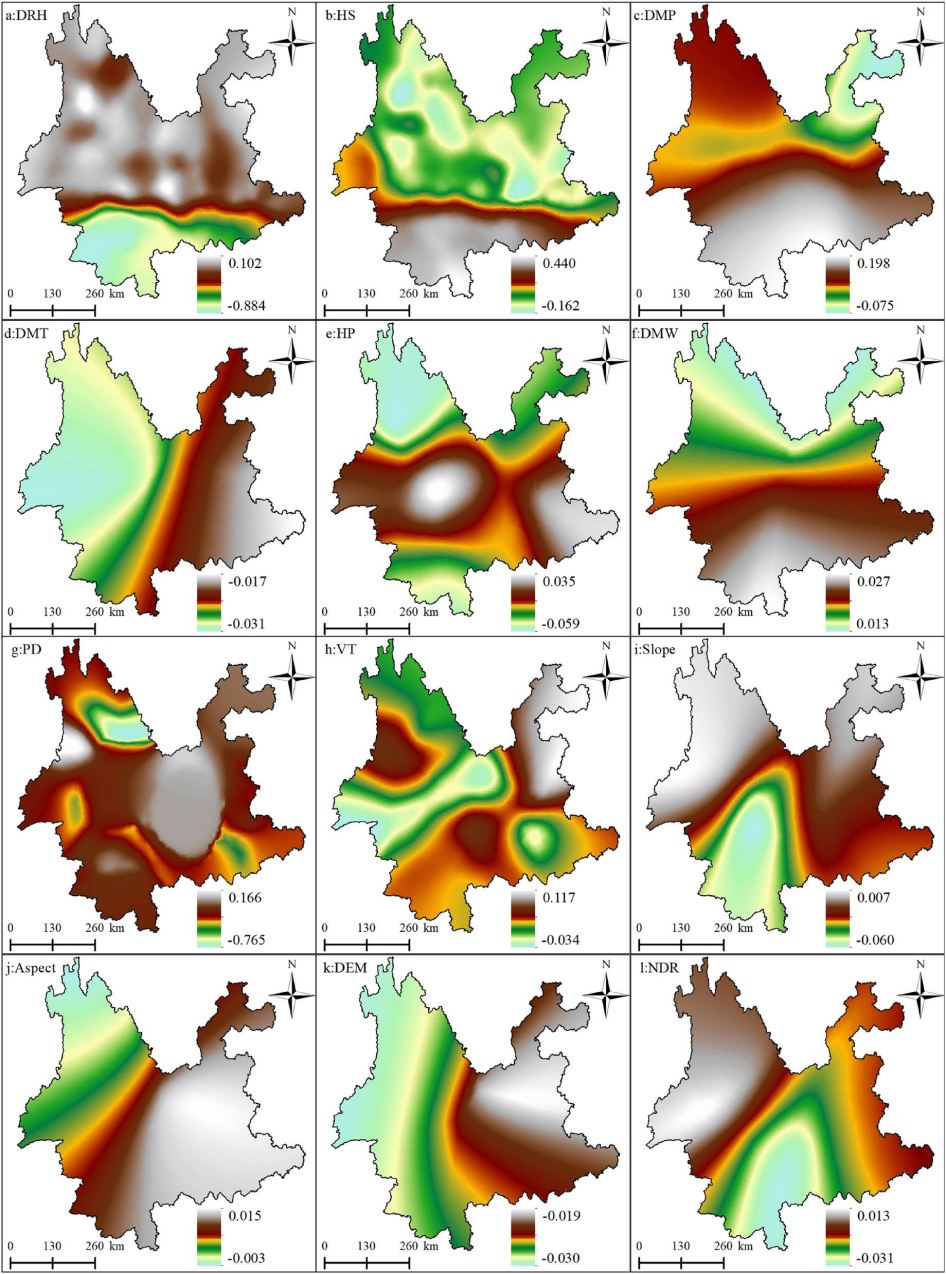
Spatial pattern of regression coefficients for each explanatory variable in the MGWR model. (**a**) Average relative humidity; (**b**) 24-h sunshine; (**c**) daily maximum pressure; (**d**) daily maximum temperature; (**e**) 24-h precipitation; (**f**) daily maximum wind speed; (**g**) population density; (**h**) vegetation types; (**i**) slope; (**j**) aspect; (**k**) digital elevation model; (**l**) nearest distance from railway to fire point. Maps were generated by ArcGIS 10.8.12790 (https://www.esri.com/).

In the MGWR, the larger the bandwidth, the slower the weight decays with increasing distance; the smaller the bandwidth, the faster the weight decay with increasing distance. Bandwidth is a sphere of influence, which is the spatial scale of the effect of the driver on forest fires. Specifically, a larger bandwidth of the driver (with a larger spatial scale of effect) has approximately the same mode and intensity of action on forest fire occurrence over a larger spatial scale range with a smaller spatial gradient. Their spatial relationships with forest fires have a weak degree of distance decay and a low degree of spatial heterogeneity, and spatial relationships tend to be stable and less sensitive to spatial location. Figures 6 and 7 show that different forest fire drivers have different spatial scales of action on forest fires and also reflect differences in the spatial heterogeneity scales of the different drivers. In the present study, the spatial scales of action of the explanatory variables are classified into three spatial scales of action, global, medium, and small scales, according to the bandwidth of each driver, which affects the probability of forest fire occurrence. Similarly, the spatial heterogeneity levels of the explanatory variables were classified into three levels: global, medium, and high heterogeneity.

In the MGWR regression results, the topographic factors of elevation and aspect, with scales of 8030 and 7992, respectively, can be considered global spatial scales of action, while the spatial distribution of the coefficients according to Fig. 7j, k is also relatively stable, meaning that there is little spatial heterogeneity, indicating that the effects of elevation and aspect are almost uniform from one fire site to another. The scale of action for the slope is 4105, which can be considered a medium scale. Simultaneously, the driver influences the occurrence of forest fires at a moderate level of spatial heterogeneity, which means that the distribution of the coefficient is relatively stable over space (Fig. 7i). Among all the meteorological elements, the spatial scales of action of the daily maximum temperature and maximum wind speed can be considered as global scales: 8030 and 8026, respectively. The other four drivers of forest fires were 409, 567, 3019, and 2850 for average relative humidity, sunshine hours, daily maximum pressure, and daily precipitation, respectively. Medium relative humidity and sunshine hours can be considered small-scale effects, because their effects on forest fires can be considered highly heterogeneous; the spatial scales of action of air pressure and precipitation are medium scale. The spatial distribution of the regression coefficients of these four factors in the four panels of Fig. 7b, d, e, g also shows the level of spatial heterogeneity in their effects on forest fires. This shows that among the meteorological factors, forest fires are most sensitive to relative humidity and sunshine hours. Among the anthropogenic factors, population density has an effect scale of 997, which can be considered a small-scale influence on forest fires, that is, a high level of spatial heterogeneity in its influence on forest fires, to which the occurrence of forest fires is more sensitive. The scale of action for the distance from the road to the fire site can be considered medium, with a scale of action of 5702, which also indicates a medium level of spatial heterogeneity in the effect of this factor on forest fires. The vegetation type factor had an effect scale of 1718, indicating a moderate level of spatial heterogeneity in the effect of vegetation type on the occurrence of forest fires.

The spatial scale effects of the different drivers on forest fire effects vary, with the four drivers of mean relative humidity, sunshine hours, daily maximum pressure, and daily precipitation having smaller scales of action, greater distance attenuation, and greater degrees of spatial heterogeneity than the other drivers, indicating that forest fire occurrence is more sensitive to these drivers. The other drivers range from moderate to near-global scales, suggesting that there is variability in the scale of heterogeneity of their effects on forest fires and differences in their sensitivity to them. Therefore, future studies on the spatial prediction of forest fires should consider that different drivers show heterogeneity in their scales.

## Discussion and conclusions

### Discussion

Figures 2 and 3 of the GWLR modeling results show that different drivers contribute to and influence the probability of forest fire occurrence differently, both positively and negatively, and the same driver at different spatial locations also has different effects on forest fires. Among them, meteorological factors such as sunshine hours, air pressure, temperature, and wind speed have significant positive correlations with the probability of forest fires, while relative humidity and daily precipitation have significant negative correlations with the occurrence of forest fires; vegetation type has positive effects on forest fires; the topographic factors slope, aspect, and elevation are negatively correlated with forest fires; anthropogenic activities two factors of population density and the closest distance from the road to the fire point are negatively correlated with the occurrence of forest fires.

Meteorological factors, such as sunshine hours were significantly and positively correlated with the occurrence of forest fires, which is consistent with the results of previous studies^34^. This is because the number of sunlight hours greatly affects the water content of combustible material in the forest understory; as sunshine hours increase, evaporation increases, and the combustible material in the forest understory becomes drier and more likely to reach the threshold for forest fires. Therefore, the probability of a forest fire increases as the number of hours of sunshine per day or within a given period increases; the modeling results show a significant positive correlation between air pressure and temperature and forest fire occurrence, which means that as barometric pressure and temperature increase, the probability of forest fire occurrence increases. This result is consistent with other research findings^35,36^. Temperature and air pressure are key drivers of forest fires. A gradual increase in air pressure and temperature, indicating clear weather, directly increases the rate of evapotranspiration from the trees and from combustible material in the forest, indirectly increasing the probability of a forest fire. The model also show a positive correlation between wind speed and forest fire occurrence, indicating that the probability of forest fires increases with an increase in windy weather and wind speed. The magnitude of the wind is particularly relevant to the occurrence of forest fires; not only does it rapidly increase the dryness of combustible material in the forest to help increase the probability and intensity of combustion, but it also ultimately increases the probability of forest fires. Moreover, it can provide sufficient oxygen to the fire source to accelerate combustion and expand the fire area after a forest fire has occurred. The results of the study showed a significant negative correlation between relative humidity and the occurrence of forest fires, which means that as relative humidity increases, the probability of forest fires decreases, which corresponds to the results of other studies^34^. When relative humidity increases, the humidity of the forest itself increases, and the water content of combustible materials on the forest floor, such as fallen leaves and dry weeds, increases to the extent that the probability of a forest fire is reduced. Conversely, when relative humidity decreases, the probability of forest fires is likely to increase. The modeling results showed that the probability of forest fire occurrence also decreases with increasing precipitation, which is consistent with other studies^37,38^. When precipitation decreases, the humidity of the forest itself and the moisture content of the combustible material in the forest continues to decrease and the combustible material becomes drier, leading to an increase in the probability and severity of forest fires. Conversely, when precipitation increases, it may lead to a decrease in temperature and an increase in air humidity, increasing the moisture content of the combustible material in the forest and decreasing the probability of forest fires.

**Figure 8:**
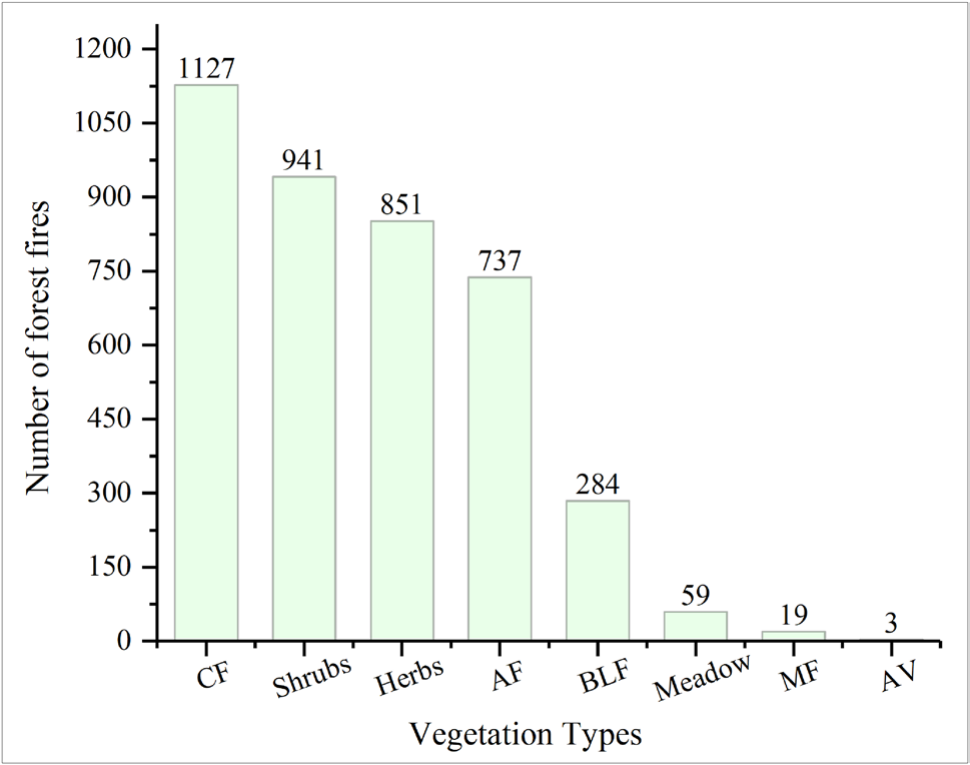
Number of fires in each vegetation type in 2010–2020. Coniferous Forest (CF); Shrubs; Herbs; Artificial Forest (AF); Broad-Leaved Forest (BLF); Meadow; Coniferous and Broad-Leaved Mixed Forest (MF) ; Alpine Vegetation (AV).

MGWR modeling results show a positive effect of vegetation type on forest fires, which is consistent with other studies that suggest that vegetation types such as eucalyptus, coniferous forests, and scrub contribute to the probability of forest fires^39^. This indicates that the main vegetation types in the Yunnan Province, such as Yunnan pine and planted eucalyptus forests, may increase the probability of forest fires. As can be seen from Fig. 8, the vegetation types occupied by the forest fire sample sites were highest in coniferous forests, followed by shrublands, grasses, and plantation vegetation. Therefore, the probability of forest fires increases in spatial locations with a large distribution of these vegetation types^40^.

Topographical factors, such as slope and altitude, have a negative effect on forest fires. Altitude affects humidity. The higher the altitude, the lower the temperature and the higher the relative humidity in the forest interior. Ground cover plants have increased water content and are less likely to burn, whereas at lower altitudes, the opposite is true. Therefore, the probability of forest fires decreases as altitude increases. Slope gradient directly affects the rate of change of water content of combustible material. A steep slope easily loses water, leaving combustible material dry and flammable, and the probability of forest fire increases; in contrast, when the slope moisture retention time is long, the forest floor is wet, reducing the probability of forest fire^41,42^. The effect of aspect on forest fires is generally more sensitive on southern slopes than on northern slopes because southern slopes receive more sunlight than northern slopes, resulting in lower water content and drier vegetation. Therefore, the probability of forest fires on southern slopes increases. At the same time, the distribution of the absolute values of the coefficients in Fig. 3j shows that the influence of the slope direction on forest fires tends to increase from north to south.

Of the anthropogenic factors, population density has a negative effect on the occurrence of forest fires. That is, as population density increases, the probability of forest fires decreases. The reason for this phenomenon is likely to be that the more densely populated areas are more urbanized, such as Kunming, Anning, and Qujing in central Yunnan, which are densely populated but have fewer forested areas, less agricultural activity, and a more concentrated firefighting force. Second, the densely populated locations in rural areas are often near residential areas where people are more aware of the protection of their living environment. Therefore, the population density negatively influences the occurrence of forest fires. The distance from the road to the fire point has a negative effect on the occurrence of forest fires; that is, as the distance from the road to the fire point increases, the probability of a forest fire decreases, which is consistent with the results of previous studies^43–45^. The closer the road is to the forest, the more human activity will be associated with forest fires, such that the likelihood of anthropogenic fires is increased.

According to Figs. 6 and 7, the results of MGWR modeling show that the spatial relationship between forest fires and drivers has obvious spatial non-stationarity; there is some variability in the spatial scales of action of different drivers on forest fires. Among the meteorological factors, mean relative humidity, sunshine hours, daily maximum pressure, and daily precipitation, had smaller scales of action (smaller bandwidth) than the other drivers. This suggests that forest fire occurrence is more sensitive to these drivers and that the spatial non-stationarity between them and forest fires is more pronounced, reflecting stronger spatial heterogeneity (Fig. 7a–c, e). The main reason for this is that the spatial scale of the study area is large, so most of its meteorological factors are spatially distributed with variability and imbalance, leading to a smaller spatial contribution to forest fires. For example, the extent and amount of precipitation vary considerably in spatial distribution, which means that rainfall itself tends to be localized in spatial distribution and therefore has a smaller impact on forest fires. The spatial scales of action of the topographic factor elevation and aspect are almost global, whereas the spatial distribution of the coefficients according to Fig. 7k, g is relatively uniform, meaning that there is little spatial heterogeneity. The slope has a medium scale of action, which means that there is a medium level of spatial heterogeneity. The reason for this is that the effect of slope direction on forest fires is mainly on the north and south slopes, as the southern slopes are more susceptible to forest fires than the northern slopes, so the effect of aspect tends to be more stable than the other factors, and is spatially global in scale. The effect of elevation on forest fires also tends to be global in scale, because the occurrence of forest fires in Yunnan Province is spatially aggregated, so the scale of the effect of elevation is less variable. However, the moderate effect of slope is due to the spatial heterogeneity of the undulating and uneven topography there, and therefore, the effect of slope on forest fires tends to be more local in scale. Among the human activity factors, population density had a small-scale effect, and the distance from the road to the fire site had a medium-scale effect on forest fires. The spatially uneven distribution of population density leads to considerable local variation in its effect on forest fires, whereas the distribution of roads is more regular and therefore has a relatively large-scale effect on forest fires.

**Figure 9:**
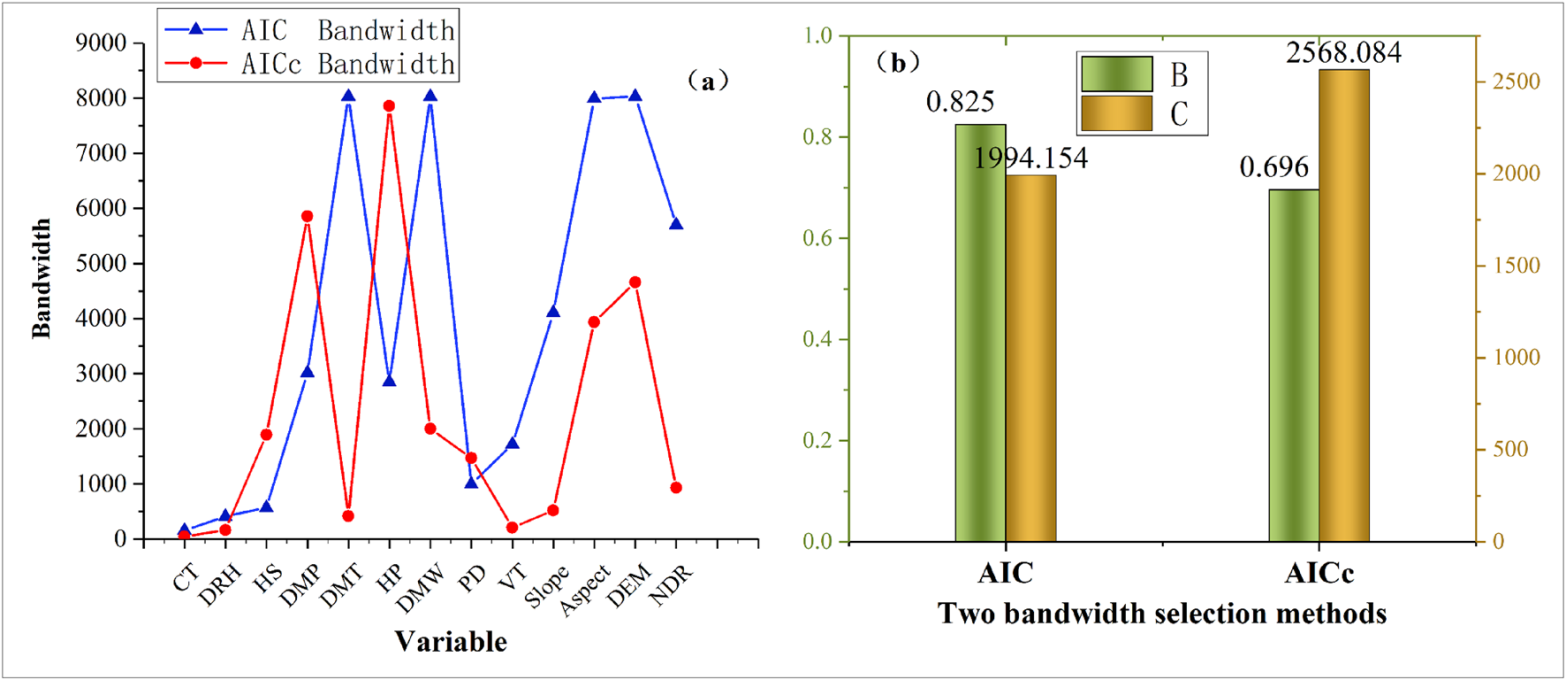
Fitting effect of AIC and AICc. (**a**) Determination of bandwidth based on AIC and AICc: constant term (CT); Daily relative humidity (DRH); 24 h of sunshine (HS); daily maximum pressure (DMP); daily maximum temperature (DMT); 24-h precipitation (HP); daily maximum wind speed (DMW); population density (PD); vegetation types (VT); slope; aspect; digital elevation model (DEM); nearest distance from the road to fire point (NDR); (**b**) B shows the model fit of R^2^, C is the sum of squared residuals.

The bandwidth size directly determines the range of valid data points around each regression analysis point in the MGWR model-solving process, which is the spatial scale of action of the driver. Moreover, a bandwidth that is too small may lead to local overfitting of the model and dramatic spatial variation in parameter estimates, which indicates that the scale of action of the driver tends to be global and parameter estimates tend to be spatially smooth; that is, the scale of spatial heterogeneity of the factor is small. Therefore, selection of a suitable bandwidth is necessary. For the MGWR model, the bandwidth value can be selected using either the Akaike information criterion (AIC) or the modified Akaike information criterion (AICc). In the present study, the better bandwidth method was selected by comparing the R^2^ of the fit and the sum of squared residuals of the fit (Fig. 9). Based on the results in this figure, the AIC method has better R^2^ and residual squared values.

### Conclusions and further efforts

The present study combines the GWLR and MGWR models to identify the main drivers of forest fires and to explore the mechanisms by which they influence the probability of forest fires. First, based on the forest fire monitoring data of the Yunnan Province from 2010 to 2020, two types of data were combined: natural environmental (meteorology, topography, and vegetation type) and anthropogenic data (population density, nearest distance from road to fire). A geographically weighted logistic regression model was used to model the main forest fire drivers and to predict the probability of forest fire occurrence at each location. Second, the probability of forest fires and identified drivers were combined with the MGWR model to detect the effect and scale of action of each driver on the occurrence of forest fires. The results of the GWLR modeling show that meteorological factors such as relative humidity, air pressure, temperature, precipitation, and sunshine hours; anthropogenic data such as population density and distance of roads from fire points, topographic factors such as slope, elevation, and slope direction, and vegetation type are the drivers of forest fires in the Yunnan Province. Then, the results of MGWR modeling showed that different drivers have different scales of action on forest fires, and the spatial scale effects of different drivers vary greatly. For example, the spatial effects of topographic factors on forest fires tend to be on a global scale, whereas some meteorological factors tend to be on a local scale, and show spatial non-stationarity between drivers and forest fires. Based on the results of this study, it is suggested that the spatial scales of action of drivers on forest fires are different, and future studies on spatial prediction of forest fires should take this into account. This will further improve the accuracy and make prediction results more realistic. This study showed that combining the GWLR and MGWR models to detect their spatial scales of action is useful for analyzing the mechanisms by which drivers influence forest fires. However, this study had a limitation. We could not combine the MGWR model with existing forest fire data-driven models to make spatial predictions of forest fires based on the detected mechanisms of driver effects on forest fires; therefore, this aspect can be an important topic for further research. In the next stage of investigation, we intend to combine the MGWR model with a binary logistic regression model and apply it to research on spatial prediction of forest fires, identification of drivers, and analysis of the spatial relationship between forest fires and drivers.

## Materials and methods

### Study area

**Figure 10:**
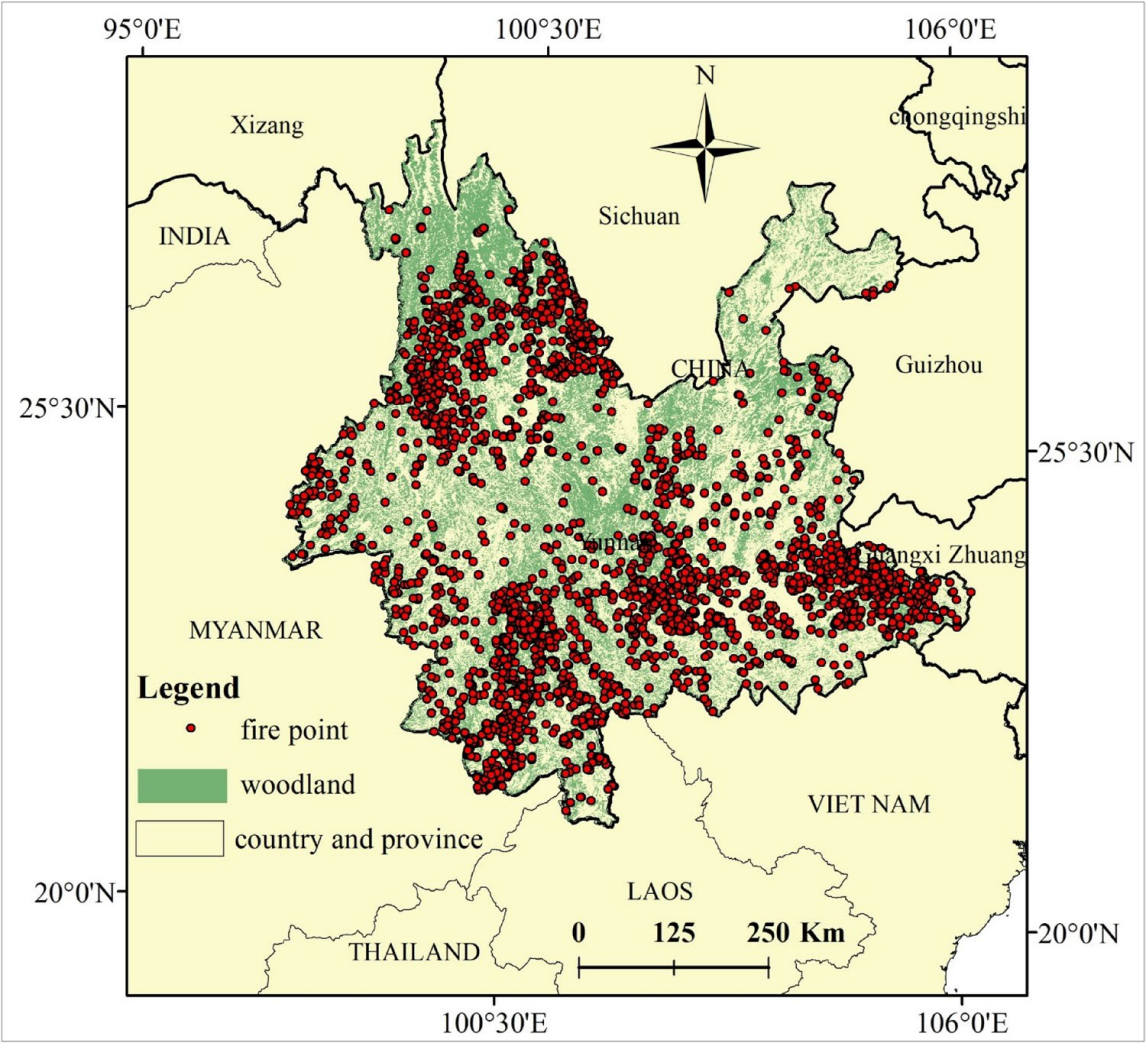
Study Area, Yunnan, China. Maps were generated by ArcGIS 10.8.12790 (https://www.esri.com/).

Yunnan Province is located at low latitude (21°8′32″–29°15′8″ N, 97°31′39″–106°11′47″ E) on the Yunnan–Guizhou Plateau (Fig. 10), with a high northwest and low southeast terrain (maximum altitude 6740 m, minimum 76.4 m). The temperature change caused by this large altitude difference results in dramatic climate variations that are conducive to the growth of a large variety of vegetation types. This makes the Yunnan Province particularly rich in forest resources. It is a key forest area in China, with a coverage ratio of 55.7%. Yunnan Province is also the main location for forest fires in China. The dominant species belong to coniferous forest vegetation and are flammable; these include *Pinus yunnanensis*, *Pinus armandii*, *Cunninghamia lanceolata*, *and Pinus kesiya*. In recent years, artificially planted base timber forests, aerial-sown forests, and Yangtze River shelter forests contain mostly pine, fir, and eucalyptus, which are also flammable species. In addition, the monsoon climate in the Yunnan Province is distinctive, with dry seasons and high-temperature wet seasons. Precipitation during the dry season (November–April) accounts for only 15% of the annual precipitation and is unevenly distributed temporally and spatially. Spring and winter are high fire risk seasons in most areas of the Yunnan Province. The combination of flammable tree species and seasonal dry climate conditions makes Yunnan Province prone to frequent forest fires that are difficult to prevent. This is exacerbated by the diversity and complexity of flammable vegetation, dominance of flammable tree species, topography, climate environment, forest distribution, production, and domestic fires in the Yunnan Province.

### Data sources and processing

The data required for this study consisted of two main categories (as shown in Table 4): historical fire record data for different time periods, and forest fire drivers selected based on the experience of previous studies. The historical fire data are used as the dependent variable of the model in the modeling analysis, and the drivers data are the independent variable, used to identify the forest fire drivers in the region through modeling analysis using predictive models and to explore the spatial scale effects of each driver.

**Table 4:** Research data.

Variable		Data	Source	classes	Max	Min	Mean	Spatial resolution /Units
Forest fire record point (Dependent variable)		2011–2020 Historical Forest Fire Records	NASA fire information for Resource Management System (FIRMS) https://firms.modaps.eosdis.nasa.gov					
Impact factor (Indepen-dent variable)	Weather factor	temperature	China National Meteorological Data Center http://data.cma.cn/	Continuous variable	425	− 10	255	0.1 ℃
		pressure			10,001	5423	8480	0.1 hPa
		relative humidity			1000	120	610	1%
		wind speed			357	20	65	0.1 m/s
		precipitation			1306	0	67	0.1 mm
		sunshine duration			128	0	75.4	0.1 h
	Terrain factor	altitude	China Geospatial Data Cloud http://data.cma.cn/	Continuous variable	4556	0	1973	1000 m
		slope			86	0	23	
		aspect		Continuous variable	360	180	− 1	
	Vegetation factor	vegetational type	Resource and Environmental Science and Data Center, Chinese Academy of Sciences https://www.resdc.cn/	Categorical variables				1000 m
	Anthropo-genic factor	population density	Open spatial demographic data and research https://www.worldpop.org/	Continuous variable	22,210	0	62	1000 m
		Distance from road to fire	Open street map https://www.openstreetmap.org/	Continuous variable	0.532	0.001	0.027	

#### Dependent variable: forest fire data

Historical fire data for the different time periods under study were obtained from NASA's Fire Information for Resource Management System (FIRMS, https://firms.modaps.eosdis.nasa.gov/), a dataset containing active fire pixels from the Moderate Resolution Imaging Spectroradiometer (MODIS) and Visible Infrared Imaging Radiometer Suite (VIIRS) products. The present study used near real-time fire products distributed from the MODIS on the Terra and Aqua platforms. Each location of an active fire identified by MODIS represents the center of a 1 × 1-km pixel that is algorithmically tagged as containing one or more fires within the pixel. The dataset includes the latitude and longitude coordinates of the fire, date and time of the fire, confidence level of the fire, and type of fire. In order to ensure the reliability of the historical forest fire data obtained, the following three conditions were used to filter the acquired data: first, only fire data with the fire type "vegetation fire" were selected; secondly, fire data with a fire confidence level greater than 85 were selected; finally, the spatial location of the fire data was overlaid with the land-use type data to remove fire points falling on wetlands, urban land, agricultural land, and other land types. A total of 4021 fires were selected with high reliability.

The prediction model used in this study was a binary model, in which the dependent variable is of two types, 0 and 1, and is not a continuous value. In its application in the present study, 0 represents non-fire point data (no forest fire location observed) and 1 represents fire point data (clear occurrence of forest fire data). Therefore, a certain proportion of non-real fire data is required for modeling when analyzing the probability of forest fire occurrence and determining forest fire drivers in Yunnan Province. In this study, the ratio of real fire records to the fire data created for modeling was set to 1:1 based on previous research experience, which means that there were 4021 real fire record points and 4021 artificially created non-real fire points, which constituted 8042 forest fire data points in the Yunnan Province. The random creation of non-real fire data follows two rules: they must fall in areas where the land use type is forestland, and must be random in time and space.

#### Forest-fire impact factors

The drivers used in the present study included both natural environmental and anthropogenic data^46,47^. The former included meteorological, topographic, and vegetation factors. Meteorological factors, including temperature, relative humidity, sunshine hours, barometric pressure, wind speed, and daily precipitation^48–50^, were used to create Thiessen polygons of weather stations in ArcGIS10.8 based on the weather station data of the study area. Then, the 8042 identified forest fire sample points were matched with the weather station points, based on the spatial location, the weather station to which each sample point belongs was obtained, and Python was applied to match the sample points to the weather data values based on the weather station points and the date of the sample points. Topographic factors, including elevation, slope, and aspect, were obtained based on digital elevation model (DEM) data analysis^51,52^, The DEM data, which have a spatial resolution of 1 km, were downloaded from the China Geospatial Data Cloud website. Vegetation factors included only vegetation-type data^34,53^, according to the Chinese National Vegetation Classification Standard; the secondary classes of vegetation-type data were reclassified into primary classes through ArcGIS 10.8, mainly coniferous forests, broad-leaved forests, shrublands, grasslands, meadows, alpine vegetation, cultivated vegetation, and mixed coniferous and broad-leaved forests. These data were obtained by: (1) extracting the vegetation type corresponding to the sample point; (2) calculating the proportion of each vegetation type to the total vegetation type there; and (3) replacing the vegetation type of the sample point with the proportion value of the vegetation type^54^. Anthropogenic data included population density data^45,55^, road network data^52,56^. All sample point data were analyzed by overlaying them with the corresponding population density raster data through ArcGIS10.8 and the value extraction-to-point tool was applied to extract the population density value for each forest fire point, for which the road network data were calculated by applying the nearest neighbor analysis tool in ArcGIS10.8 to the nearest road for each sample point. Finally, all drivers were normalized by Eq. (1) to eliminate differences in scale, rank, and data level between the data.

1 $$\chi_{{\text{i}}} = \frac{{\chi - \chi_{\min } }}{{\chi_{\max } - \chi_{\min } }}$$

In this equation, $\chi_{{\text{i}}}$ is the normalized value, $\chi_{\max }$ is the maximum value in the dataset for a particular type of driver, and $\chi_{\min }$ is the minimum value in the dataset for a particular type of driver.

### Research process

**Figure 11:**
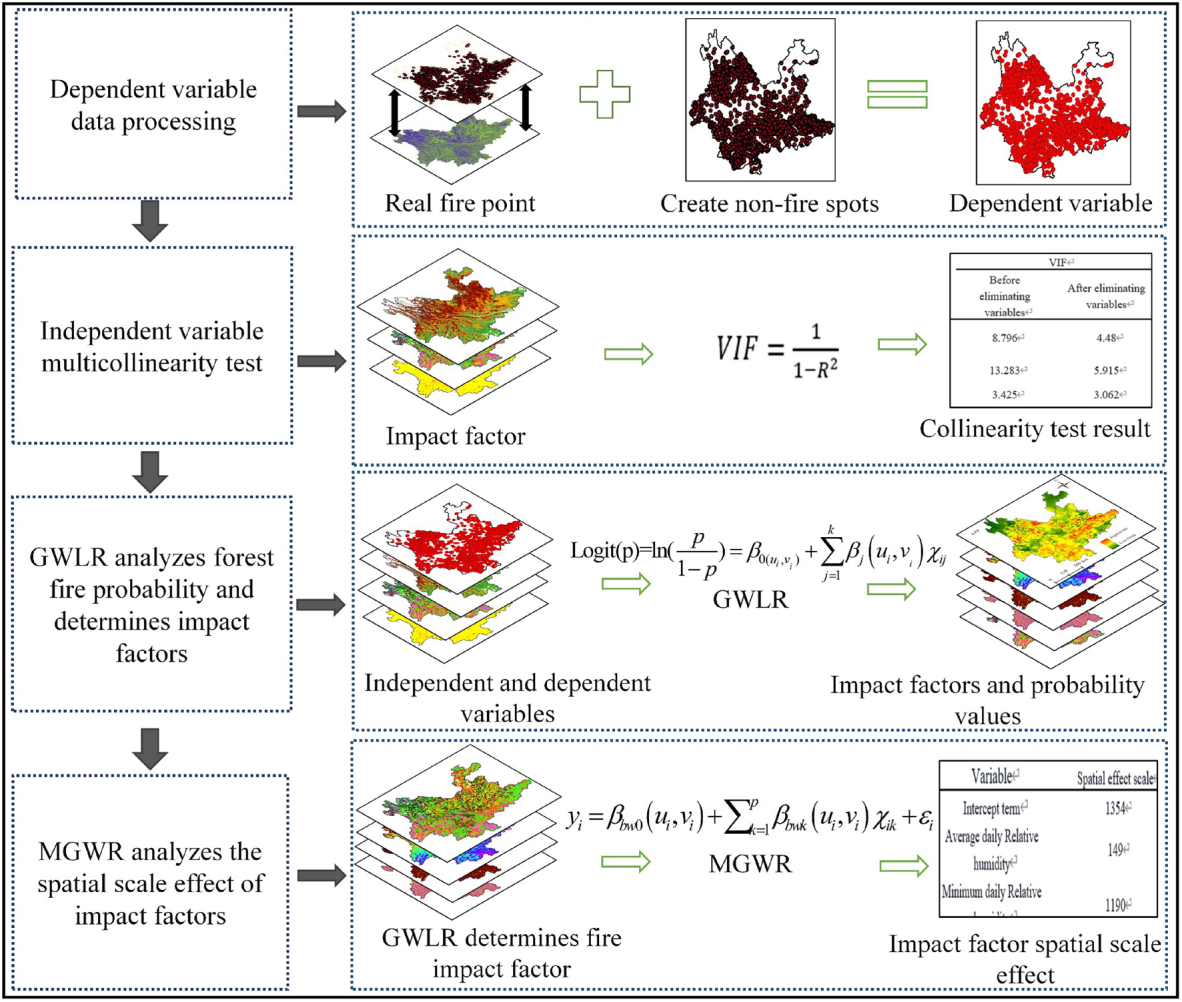
Research process.

Figure 11 is a flow diagram for the present study. First, the actual forest fire data points were extracted by overlaying the land use type data with the historical fire record data, and because the GWLR model is a binary model, the modeling required the creation of the same proportion of non-real fire data as the historical fire record points, which together formed the dependent variable; the driving factors determined from the previous study were constructed by multiple covariance testing. Second, the GWLR model was used to estimate the probability of future forest fires at spatial locations within the study area and to identify the main drivers of forest fires in the study area (in this step, the experimental data were divided into 60% training data and 40% test data for three experiments to avoid chance in the model fitting effect, and finally, the full sample data were used for modeling). Finally, the MGWR was used to detect the probability of forest fires at spatial scales. In this study, the geographic data were processed using ArcGIS10.8 software; the multicollinearity test was implemented by SPSS25.0; all the geographic interpolation spatial visualization was implemented by the Kriging interpolation tool in ArcGIS10.8 software; the fitting of GWLR and MGWR models was implemented based on MGWR2.1 software^57^.

#### Methodology

##### Multicollinearity test of variables

Multicollinearity refers to the existence of a certain degree or a high degree of correlation between the explanatory variables in a linear regression model, which can lead to the loss of significance of the variables and failure of the predictive function of the model. In this study, the variance inflation factor (VIF) was used to test for multicollinearity in the explanatory variables selected for the study, and to determine the final independent variables entering the model. The formula is as follows:

2 $$VIF = \frac{1}{{1 - R^{2} }}$$

This coefficient was interpreted by using 10 as the critical value. Multicollinearity was not observed when VIF < 10. When 10 ≤ VIF < 100, multicollinearity was high. When VIF ≥ 100, severe multicollinearity exists^53,58^.

##### Geographically weighted logistic regression

Geographically weighted logistic regression (GWLR) is an extension of the traditional logistic regression model, where the spatial location of sample points is introduced into the modeling. The effect of spatial non-smoothness between forest fires and drivers was also considered. The phenomenon of variation in the relationship or structure of variables owing to differences in geographical location is called spatial non-stationarity. The model uses weighted least squares to estimate the parameters for each sample point, and the parameter estimates were local rather than global, with corresponding parameter estimation coefficients for each location^52,59^. The expression for the geographically weighted logistic regression model (GWLR) has a probability of forest fire occurrence (Y = 1) at location i as p, and the probability of no forest fire occurrence (Y = 0) as (1−p). The regression equation between the probability of forest fire occurrence at location i and each variable X (I = 1,2,…,n) is as follows:

3 $$P(Y = 1)\frac{{\exp^{{\left( {\beta_{{0(u_{i} ,v_{i} )}} + \sum\limits_{j = 1}^{k} {\beta_{j} } \left( {u_{i} ,v_{i} } \right)\chi_{ij} } \right)}} }}{{1 + \exp^{{\left( {\beta_{{0(u_{i} ,v_{i} )}} + \sum\limits_{j = 1}^{k} {\beta_{j} } \left( {u_{i} ,v_{i} } \right)\chi_{ij} } \right)}} }} = \frac{{e^{ - z} }}{{1 + e^{ - z} }} = \frac{1}{{1 + \mathop e\nolimits^{ - z} }}$$

Among these,

4 $${\rm Z} = \beta_{{0\left( {u_{i} ,v_{i} } \right)}} + \sum\limits_{j = 1}^{k} {\beta_{j} } \left( {u_{i} ,v_{i} } \right)\chi_{ij}$$

The GWLR model obtained by logical transformation is as follows:

5 $${\text{Logit(p) = ln}}\left( {\frac{p}{1 - p}} \right) = \beta_{{0(u_{i} ,v_{i} )}} + \sum\limits_{j = 1}^{k} {\beta_{j} } \left( {u_{i} ,v_{i} } \right)\chi_{ij}$$

where (*u*_*i*_*,v*_*i*_) are the geographical coordinates of fire point *i*; *X*_*i1*_*,X*_*i2*_*,…, X*_*ij*_ are the independent variables; and *Z* is the estimated value of the estimated coefficient of the local regression model for location *i*.

The bandwidth is an important control parameter for the GWR model, because it directly determines the rate of weight decay with increasing distance. The greater the bandwidth, the slower is the weight decay, and vice versa. The bandwidth-selection methods of the GWR model are adaptive and fixed. The GWLR model selects an adaptive bandwidth by defining the number of nearest neighbors *M*. The distance between the sample point *i* and the nearest neighbor *M* is used as the bandwidth, where *M* is determined by the Akaike information criterion (AIC) (Eq. 6), and the size of *M* can directly represent the size of the bandwidth^60,61^.

6 $$AIC = 2K - 2\ln (L)$$

where *L* is the maximum likelihood function of the model, *n* is the number of data samples, and *k* is the number of parameters of the model.

##### Spatial-scale effect of impact factors-MGWR

In the present study, the probability of forest fire occurrence and the forest-fire impact factor obtained by the GWLR model were used as the dependent and independent variables, respectively, and the spatial-scale effect of each forest-fire impact factor was analyzed in combination with the MGWR model. Traditional regression analysis assumes that all impact factors affect the relationship between forest fire occurrence and impact factors on the same spatial scale. The MGWR abandons this assumption, allowing each variable to establish a relationship with its optimal bandwidth and dependent variables^28,62^, The MGWR equation is as follows:

7 $$y_{i} = \beta_{bw0} \left( {u_{i} ,v_{i} } \right) + \sum\nolimits_{k = 1}^{p} {\beta_{bwk} } \left( {u_{i} ,v_{i} } \right)\chi_{ik} + \varepsilon_{i}$$

where *(u*_*i*_*,v*_*i*_*)* are the geographic coordinates of fire point *i*, $\beta_{bw0}$ and $\beta_{bwk}$ are the intercepts under the optimal bandwidth and the regression coefficient of the *k*-th index, respectively, *p* is the number of selected impact factors, $\chi_{ik}$ is the value of the *k* impact factors at position *i*, and $\varepsilon_{i}$ is the random error. Because of the uneven spatial distribution of sample points in the study area, this study selects the adaptive bandwidth by defining the number of nearest neighbors *M*; the distance between the regression analysis sample point *i* and the nearest neighbor of *M* is used as the bandwidth. *M* is determined by AIC (Eq. 6), and the size of *M* intuitively characterizes the size of the bandwidth^28,62^. The bandwidth size directly determines the rate at which the weight decays with increasing distance. The larger the bandwidth, the slower is the weight decay, and vice versa. Therefore, bandwidth can reflect the scale of action of the independent variable on the dependent variable^63–67^, the effect of the impact factor, and account for changes in spatial scale effects.

## Data Availability

The datasets used and/or analyzed during the current study are available from the corresponding author on reasonable request.
